# Examining the resilience of mobile youth in KwaZulu-Natal, South Africa: a qualitative inquiry through the lens of protection and risk

**DOI:** 10.1186/s12889-025-22675-7

**Published:** 2025-04-23

**Authors:** Nothando Ngwenya, Sarah Bernays, Nondumiso Dlamini, Maryam Shahmanesh, Janet Seeley

**Affiliations:** 1 Africa Health Research Institute, KwaZulu-Natal, Somkhele, South Africa; 2 School of Nursing and Public Health, University of KwaZulu-Natal, Durban, South Africa; 3 Sydney School of Public Health, University of Sydney, Sydney, Australia; 4 Department of Global Health and Development, London School of Hygiene and Tropical Medicine, London, UK; 5 Institute for Global Health, University College London, London, UK

**Keywords:** Youth, Mobility, Health, Uncertainty, Migration, Resilience, Vulnerability

## Abstract

**Background:**

The last few decades have seen a demographic shift in the age of migrants with more young people involved, especially with regards to internal migration. Socio-economic deprivation, limited resources and adversities contribute to many young people leaving rural areas in low- and middle-income countries in search of a better life in urban settings. This move often requires an adaptation process and resilience to the adversities young people face while still in the challenging developmental life stage of adolescence, exposing them to health and physical risks.

**Methods:**

As part of Lending a Hand support intervention, we conducted repeat in-depth interviews with 20 young people that had recently relocated from other rural villages to a peri-urban setting in uMkhanyakude district, KwaZulu-Natal in South Africa. Data were analysed thematically using inductive and deductive approaches and managed in NVivo software.

**Results:**

The participants shared that there were alarming rates of teenage pregnancies in their local area and attributed this to younger girls dating older men for economic gain, which also exposed them to infectious diseases. Some vulnerabilities described by young people included coming from a single parent home, experiencing psychological distress, and living far away from the support of family. Other young people were able to use community-based resources as well as adaptive emotion regulation strategies that offered protective support such as church, school, and peer networks around them.

**Conclusions:**

The accessibility and availability of protective resources offered support and provided strength to young people. This fostered resilience for these young people and in a way incorporated aspects of the collectivist communities they live in. Considering resources that are easily available in resource limited settings is important as young people may be more comfortable and confident to access and use. These protective factors may help curb some of the impact of the risks that they are exposed to.

## Background

Africa is described as the youngest continent because of a bulging youth population with 60% of the population being under the age of 25 years [1, 2]. These young people experience enormous challenges due to the adversities and obstacles they encounter, which compel some of them to migrate [3, 4]. For many young people in lower- and middle-income countries (LMIC), migration is a part of the transition to adulthood mostly for education and employment opportunities [5]. Partly due to urbanization, labour migration has become more frequent, which has seen a rise in rural-to-urban migration [6]. This phenomenon is apparent in South Africa and exceeds cross-border movement, with the last national census in 2011 reporting an in-country movement of 5% of the population [7].

For young people, the adversities posed by migration are compounded by cognitive, emotional, and interpersonal changes during the developmental stage of adolescence [8]. It is during adolescence that the influence of parents and family is gradually replaced by that of friends, peers, and other external forces such as media [5]. Other key transitions during this life stage include the development of personal agency, engaging in romantic relationships, experiencing sexual debut, completing education, entering employment, leaving home and, for some, migrating to cities or across borders in search of better opportunities [5]. It is also during this developmental stage that the onset of mental health disorders such as depression and anxiety may emerge, with poor diet, and other lifestyle issues also contributing to mental and behavioural health problems [8, 9]. This increases the vulnerability of adolescents where additional stressors may lead to worse outcomes for their mental health.

Although there is an abundance of literature documenting the experiences of youth who migrate and the challenges they experience in relation to health, social status and uncertainty, there is less research examining resilience in the face of adversities posed by internal migration. Resilience is broadly defined as the process of positive adaptation to a stressor or adversity [10]. Whatever the reason for mobility, migration is recognised as a determinant of health, impacting mental health and contributing to health inequities, leading to poor health outcomes [11–14].

Youth migrants may experience internalised disorders such as depression and anxiety as they encounter various forms of adversity that may impair their identity construction [15]. Sometimes they experience a sense of disconnection from their families and communities in the new environment, especially when cultural differences, a lack of social support and difficulties integrating create challenges even if in the same country.

Circular migration is quite common in South Africa, moving between the urban areas for work and rural areas where there may be a permanent home [7]. Even for those who move back and forth between a rural home and urban workplaces, there are factors which affect their health, the stress of adjustment when relocating, and the disruptive effects of moving from one place to another, as well as managing existing health conditions in a new place. Exposure to violence, insecurity and exploitation are risks young migrants can face [16]. Securing safe accommodation, making friends, and accessing reliable sources of information and support can provide a buffer against these stressors, helping young people build resilience as they adapt to their new environment.

### Conceptual framework

This study examined the concept of resilience using the ‘protection-risk’ conceptual framework to guide data collection and to analyse health seeking behaviours of young mobile people in rural KwaZulu-Natal, South Africa [17–19]. Protective and risk factors are essential constructs in conceptualising resilience. Resilience within youth migration provides a suitable lens with which to understand the factors that lead to positive adaptation and development of young people facing adversity which are important for their wellbeing [20]. Resilience is defined as “the process of overcoming negative effects of risk exposure, coping successfully with traumatic experiences, and avoiding negative trajectories associated with risk” [21]. Our use of the concept of resilience aims to understand the factors that contribute to young people’s ability to adapt to the adversities they face during migration as well as the processes and mechanisms that facilitate this adaptation. One way to look at this would be identifying the protective and risk factors to know how young people cope with adversity. Advances in resilience research have indicated a considerable difference in the adaptation process of youth migrants and youth experiencing other adversities and traumatic events [22–24]. The model used for this study is the protection-risk framework developed by Kabiru and colleagues. This framework uses three types of risk factors (models’ risk, opportunity risk, vulnerability risk) and three types of protective factors (models protection, controls protection, support protection) [17]. Risk describes factors that lead to a problematic event, while protective factors modify risk and reduce the likelihood of the problematic event. Models of risk refers to role models that may negatively influence young people to engage in risk behaviours. Opportunity risk includes situations or environment such as working in a bar that inevitable exposes a young person engaging in risk behaviours. Vulnerability risk are the internal individual factors that increase chances of engaging in risky behaviours such as hopelessness. Models of protection are role models who unlike models of risk, promote prosocial behaviour. Controls protection are individual level or environmental factors that serve as regulatory controls and thus deter risk behaviours. Support protection includes contextual factors such as peer networks that promote health enhancing behaviours. All these factors intersect with both risk and protective factors that influence the acculturation experiences of young mobile people affecting their adaptation to their new environment and impacting their health and mental wellbeing. However, sometimes these young people can be resilient in the face of adversity which is an important factor in understanding and identifying the pathways that lead to this positive adjustment.

The concept of resilience has been described as both a process and an outcome [25–27]. Adversity (risk), outcomes and mediating factors are essential interconnected components to be investigated in resilience research, moving beyond observation of process to explaining the outcomes [27]. This paper presents findings on sources of resilience among young migrants, offering a conceptual understanding of young mobile people’s needs and practices that can support positive adjustment. Explaining the phenomenon of resilience in the context of adversity is important in learning how problems are averted and how good outcomes are achieved by young people at risk.

## Methods

The Lending a Hand project was a study designed to assess the acceptability and feasibility of a protective support structure for young migrants (aged 14–24 years old) in urban settings in South Africa and Uganda. The project aimed to provide early intervention to mitigate risks associated with youth migration, by providing peer to peer support from other young people with migration experience (peer supporters) [19]. This paper is based on the qualitative data from the South Africa site only.

The study in South Africa was conducted from May 2022 to October 2022 in a peri-urban setting in uMkhanyakude district of KwaZulu-Natal, one of the poorest districts in South Africa. In this area, migrants from rural areas relocate to peri-urban areas for education and to look for work [28]. Our focus was on young people who had migrated within six months of their move to the new location. Repeat in depth interviews (IDI) were conducted with twenty young people. The purpose of the IDIs was to collect narratives of the young people’s experiences of daily life, mobility patterns and their reasons, including mobility and income earning experiences, and effective harm reduction support they were aware of or had received. Repeat interviews were used to enrich the data generation by capturing changes that were occurring in the young person’s life as well as to nurture trust between the interviewer and interviewee. During follow-ups, 4 interviews were conducted over the phone as some participants had relocated, and other interviews conducted at a place chosen by the participants – most often at home.

### Participants

Twenty (11 male and 9 females, mean age of 19) adolescents and young adults were involved in an in-depth face to face interview with an average duration of 50 min. Young people recruited had recently moved to the area however due to recruitment challenges we extended the time they had been in the area from six months to one year. Most of the participants were recruited with the help of the peer supporters and through the snowballing methods where other young people that had been recruited referred their friends to the study team. According to their reason for moving, the participants could be divided into two groups: labour migrants and school student migrants.

### Data collection and analysis

Informed written consent was obtained from all participants before any data collection activities and informed assent obtained from minors under the age of 18 years. All the interviews were audio recorded and transcribed into IsiZulu, the local language in the area and then translated into English. No-one was present during the interviews besides the participant and researcher. The interviews were conducted by one of the author’s (ND) a female social science research assistant with training in conducting qualitative research and a native speaker of IsiZulu. Field notes were taken during the interview and reflections recorded immediately after the interview. Transcriptions and translations were done by the interviewer and double checked by the social science department core coordinator to ensure quality.

Thematic analysis was initially manually done using Microsoft Word to identify emerging codes followed by importing to NVivo software for data management. First, transcriptions were read thoroughly to identify broad themes, by two authors (ND, SB) and then patterns were identified across the data. Next, codes were developed under the ‘protection-risk’ conceptual framework and discussed by the team (NN, ND, MS, SB led by JS) as well as issues that emerged, guided by the topic guide, until no new the information was emerging. Matrices were developed covering broad topics to reduce and summarize the data and associations across themes formed. Although the results were not shared with the participants, the overall study findings were discussed with the peer supporters to check accuracy in data interpretation and validation. Due to the sample size, comparisons across groups (age and gender) were not conducted.

### Ethical considerations

This research was performed in accordance with the Declaration of Helsinki and permission was sought and obtained from the AHRI Community Advisory Board (CAB) and approved by the University of KwaZulu-Natal Biomedical Research Ethics Committee (UKZN BREC) (BREC/00001025/2020). Written informed consent was obtained from all participants above the age of 18 years. For minors under the age of 18 years we obtained parental/guardian written informed consent and written informed assent from the participant. All data were collected by a research assistant trained in qualitative research and ethical conduct of research with adolescent participants. Participants’ confidentiality was ensured throughout the conduct of the research and data stored securely in a password protected server.

## Results

We present our findings under two broad themes of *risk factors* and *protective factors* identified using the ‘protection-risk’ conceptual framework. We move beyond observing outcomes to explain within each sub-theme the patterns of influence on these experiences promoting or hindering resilience.

### Risk factors

#### Adversity and health challenges

As young migrants described their everyday lives and reasons for their move, they expressed thoughts on risk exposure. This exposure was expressed in relation to vulnerabilities linked to their social status as mobile people. Sometimes these risks were clustered due to multiple adversities including violence, poverty in the family or coming from a single parent home and living in a disadvantaged area such as a rural setting with limited access. An 18-year-old male shared that his father passed away forcing him to move from his home to the new place he now lives. He explained the circumstances that caused the eventual death of his father who was shot and therefore it was a matter of safety for him to leave home as there was no-one to take care of him:

“*The person with whom we lived was injured and it was a person who was looking after us at home*,* I would say that was my father and we moved after that… He was attacked by people who shot him*,* due to that we moved from the area*.”

As the young person explained ‘I would say that was my father’ indicating that the man who raised him was not his biological parent. Fostering of children is common in the area, where children grow up being looked after by extended family members this may be to provide be closer to school or to reduce the burden on a parent bring up children alone because of orphanhood, abandonment or family members migrating for work.

Most of the young people described these adversities from an early age which further perpetuated the situations they found themselves in after their move. These adversities included the loss of a parent which can leave a young person distressed. Others talked about growing up without the presence of a father, because the relationship between their parents had ended, which made them feel unloved and abandoned. A 15-year-old female explained described living with mental distress, attributed to her father abandoning her when she was young.:

*“all of those problems that give me a stress*,* because my mood would change many times in one day*,* my thoughts would be many and all over*,* on the other side it is my father with regards the fact that he left me when I was young*,* he is also troubling me I see him in his absence*,* I don’t know if he is sick or not*,* it keeps on showing to me*,* all of that*.”

She had been informed that her mental distress was due to traditional spiritual causes and so she was unable to seek and get assistance from the health care facility. Within the study setting many people believe in and practice African spirituality and traditional health care methods. This may involve beliefs in supernatural forces or ancestral callings, that may be identified through symptoms similar to mental health conditions and syndromes. The occurrence of such problems that are ascribed to traditional practices can be challenging for young people who may not know how and where to access support.

We explored young mobile people’s perceptions of the major health challenges in the area, and most were quick to identify health problems associated with risky behaviours. Most of these health challenges were a result of limited resources such as employment and a lack of recreation facilities:

*“I have noticed young people who are around 12*,* 11 years of age. They are in the same situation that I am in now. When I look around*,* I see a young girl that is pregnant.*”

When asked about the reasons for high teenage pregnancies in the area, this 23-year-old female alluded to the concept that young people in developing their identity and agency may feel that they want to make their own decisions and choices:

*“Young people nowadays think they are exercising their rights. They feel like they are enjoying their youth. A young person would say they are enjoying their youth*.”

This was a major health issue in the community as another 18-year-old male expressed that within his school there were many pregnant young girls:

“But *according to my observations*,* in short*,* I would say young females fall pregnant! That is the main challenge at our school*,* most young females fall pregnant*,* and we wonder why this is happening as these people are still very young. In my class*,* it was shocking to see many females who are pregnant*.”

It should be noted that within the study setting some young people have a belief that they need to become pregnant when they are a teenager as a confirmation of their fertility and ability to bear children. This is seen to increase the worth of a woman. However, when the young man was questioned by the interviewer on the reason for these early pregnancies, he shared that young girls were dating older men in the community. This is most often for economic benefit as he explained:

“The *young males from our school are not responsible for the pregnancy. It is the young females themselves who decide to date older males… we as young males would often see some young females getting picked up by males who drive VW polo cars*,* and we talk about this. They often say that they are in the business of making money when they are dating older males.”*

#### Perceptions of vulnerability

Young mobile people are vulnerable to many negative influences including the potential for exploitation and abuse as they do not have many options or a way out of their situations. Sometimes the reasons for mobility are due to the influence of parents. A 22-year-old male described his situation of moving to a new town at the request of his father who asked him to look after his (father’s) home and yet had now abandoned him. The young man described the stress this situation causes him:

“*He goes to Cape town he forgets that he left me alone here*,* when I call him*,* he says he’s busy he will call me later and he doesn’t (call back). He took me from home*,* I live a life with stress and depression I have headache*… *I end up stressed and thinking a lot of things*… *Sometimes I ask myself why am I living but I think twice that I’m not that kind of a person who commits suicide.*”

A 15-year-old female described how she was living in isolation, since she had moved to the new place, and was experiencing mental health challenges as she described:

“*On the other side I have these voices in my head talking to me every day…sometimes I would find myself having a knife to harm myself and try to take control out of that and it’s not controllable.*”

Other vulnerabilities are associated with a lack of access to healthcare services due to not knowing the practice (e.g. opening times) of the health facilities in a new place. Although South Africa offers both public and private health care, with public care being free to citizens, the state funded public healthcare service which provides care to 71% of the population is inundated with patients and always has long queues. Young people have also reported a reluctance to attend as they say they are shouted at by the nurses especially when they have sexual and reproductive needs. Not having the necessary support while going through a crisis can be challenging as shared by a 17-year-old female who experienced a miscarriage in the early hours of the morning and had to wait to attend the clinic and see a health care professional:

“*I had a miscarriage around 1 am but went to the clinic at about lunchtime*,*…around 1 pm but went home [because of the long queue] and then returned to the clinic at night. The nurse asked me*,* “Where is the foetus”? and was shouting at me saying*,* “Why did you delay coming to the clinic”? I was not in a good frame of mind to respond to his/her questions. I could not respond but felt emotional as I was being shouted at*.”

The young female described her situation as being lonely as she had no-one to confide in and her roommate was away. She explained that no-one knew about her miscarriage. Not knowing what to do, she resorted to hiding the foetus until her roommate came back:

“*I took the foetus*,* put it in a basin*,* and kept it under my bed. Then I sent my roommate (roommate’s name) a message requesting her to come back here as soon as she could. However*,* she came back a little bit late as she had washed her school clothes and had to wait for them to dry.*”

These vulnerabilities make young people have negative feelings and experiences of abandonment compounded with the physical neglect of being alone in a new place. These challenges as described by young people are significant life events that impact everyday life and can have a negative impact on self-esteem, hope, confidence, and such individual differences that influence resilience. They can influence perceptions of a young person’s identity and leave them empty and hopeless which can impede their coping skills, thus hindering resilience.

### Protective factors

#### Family and peer social support

Participants mentioned factors that helped them deal with the challenges they encountered, and the most common factor was social support. Young people were aware that social support was necessary as a source of resilience. Most often social support was from family members, neighbours, or peers. One 22-year-old male described how he relied on his neighbour sometimes for food even though the neighbour was also living in poverty with responsibility for her grandchildren:

“This *old lady will dish up in scaftin (lunch box) and give me food. I ended up telling her not to give me food every day because she is also not in a good state to give me food every day because she has grandchildren there*,* I end up sleeping on an empty stomach*.”

Grandmothers raising children is quite common in the area, mostly due to the loss of a generation of young adults during the HIV pandemic when treatment was scarce. In addition, sometimes women go to cities for work and leave their mother or other older female relative to raise their children.

The young people who maintained links with families back home expressed a strong sense of family cohesion. These young people described how they got their source of support and assistance from their family members as this 18-year-old male explained:

“*She [his mother] found this rental room for me she also bought me things that I needed when I came here. She then told my father that she found me a place and my father asked my older uncle to borrow him a car so he could bring me here*,* he got it and he drove me here and introduced me to the owner of this house.*”

He also went on to describe that he gets support he needs from his family:

“*I never ran out of food or if I have a shortage*,* I tell my mother and my father at home then they say I must come and collect it*,* or they courier via a taxi and I collect it at the bus stop*.”

Some young people did not want to bother their family as they felt that they had to stand on their own and thought their family had many other problems to deal with.

Some of the young people identified their peers as their main source of support who they could talk to and get advice from. Most of the young people were friends with people that were also new to the place. The nearby town serves as a stopover place for young people travelling to Durban or Johannesburg for employment or education, so the migrant population is quite large with a high turnover as people come and go. This made them feel they had a shared experience, and motivated one another as they knew what they were going through as this 18-year-old male explained:

“*I have taken a step back from spending time with friends*,* but I have a good relationship with my peers from the rental place. If I need anything that they have*,* they can assist. Also*,* we motivate one another to do well*.”

#### Support structures in the community; schools and churches

Young mobile people were also able to count on people that shared the same cultural, spiritual or faith beliefs as a source of support. Quite a few of them stated that they were able to get support from the church. This was a protective support structure within their community and provided hope as well as material support:

“*I ended up going to a church I don’t go to be because one pastor invited me and told me that they give food parcels.*”

Although the young people did not mention the church in relation to their spirituality, they expressed how other church members offered support and also moral guidance. This gave them hope for the future as one 19 year old male shared:

“*Well, I would say the situation is not bad for now and I can see the light at the end of the tunnel as long as I continue putting in more effort. I would say the church is also my source of support as I can approach other church members if I encounter problems. Although I don’t like to be a burden to other people, I am happy to know that there are people I can count on. I will never lack support if I need it.*”

An 18-year-old male expressed that the school and the teachers provided the support he needed and gave him practical advice that motivated him to do better:

“*There are teachers at school who can help me. My greatest motivator is the teachers, they love me, and we have a good relationship, but I don’t know the reason behind that, maybe they see potential in me.*”

## Discussion

An objective of this study was to identify the risk and protective factors experienced by mobile young people, as well as to provide an explanation of the occurrence of risk and support/protection as described by the young people. Even though health was rarely the young people’s priority, the risk factors they mentioned increased their vulnerability to ill health especially on mental health and wellbeing [14, 29]. Even though South Africa offers free public healthcare services, limited knowledge of where and when to access care, long queues, and potentially negative attitudes from health care professionals all impacted the support young people received or perceived that they could access leading to a reluctance to attend clinics. Sometimes their lack of confidence in accessing services posed a barrier to getting the services they needed. Exposure to risky behaviours left some young mobile people vulnerable to exploitation by older people, for sexual relations for example, and increased their risk of contracting infectious diseases [30, 31]. Within the research setting, there is a problem of young people involved in what have been called `blesser, blessee relationships’ for monetary and material gain [31].

Resilience varies from one person to the other due to personal factors. Resilience is also influenced by environmental factors. Young mobile people face cumulative disadvantages within their resource limited environment that increase risk. Although the young people in this study were in similar environments at the time of the interviews, their experiences and disadvantages often begin prior to their departure from home. Sometimes these factors are the reason the young person migrates, such as parental loss and violence [32, 33]. Within the concept of resilience, these exposures are considered to be chronic adversities [27]. These disadvantages shape the experiences that the young person has in the new area with a pervasive impact on their wellbeing and expose them to exploitative conditions. Sometimes this exploitation is by a relative or family acquittance in the new area that they seek help from.

Social isolation made some of the young people feel excluded from society and their community. This is a finding consistent with other literature on youth migrants who are isolated and are socially marginalised, further exposing them to exploitative circumstances [34].

The main sources of resilience that stood out were social support, from family, peer networks and community structures like the churches and schools. Some of the young people also had their individual drive and capacity such as a commitment to attain a good education for a better future [35]. These findings show how some young people can develop strategies to cope with adversity and contribute to resilience. This was seen where young people used conscious cognitive strategies such as acceptance of their situation, positive reappraisal and positive refocusing as they chose to see ‘a light at the end of the tunnel’ [36]. These emotional regulation strategies contributed to the overall protective factors identified in the current study and indicates what really matters in the positive adaptation of mobile young people. These findings are important as studies have shown how emotional regulation is associated with mental wellbeing while maladaptive emotional regulation is linked to depression, anxiety and other risk factors such as drug abuse [37]. A positive outlook could augment a young people’s support structures to help them deal with their adversities of being a mobile young person. The complex interplay between the individual and the environment when it comes to fostering resilience means young people would need to make a concerted effort ‘to act’, using their personal agency. Other research evidence has shown that external sources will bear greater positive results if a young person has greater personal agency improving their ability to regulate their emotions [38].

Our findings show that the collectivist communities in lower- and middle-income countries have structures that can provide support to mobile young people. Relationships with churches for temporal support and not only religious or spiritual support can be central to fostering resilience in young people [39]. Even though these structures did not necessarily buffer the youth from the risk factors they described and experienced, they provided support when some needed it. For others, the church community provided motivation to keep going. There seems to be a dynamic process between the vulnerabilities and risks that young people are exposed to and the resilience in the young people; such is support are fundamental coping strategies for mobile people [35, 40]. The sources of support helped an individual feel safe and secure, which is needed during this precarious time of uncertainty.

## Conclusions

For young mobile young people, it may seem the odds are stacked against them. The disadvantages they have prior to their move are compounded by the disadvantages they experience in the new places which precipitates future disadvantages. These disadvantages, including living alone in rental accommodation made them isolated and far away from family and other support structures from kin. However, most of the young people were resilient. Community-based resources such as schools and churches are central to providing solutions in resource limited settings and can provide support and bring a sense of stability for young people on the move. Young people also had the ability to use adaptive emotional regulation strategies which influenced their wellbeing and overall coping with the adversities they faced.

## Data Availability

The datasets generated and analysed during the current study are not publicly available due to their containing information that could compromise the privacy of research participants but are available from the corresponding author on reasonable request.
